# Optic Nerve and Spinal Cord Are the Major Lesions in Each Relapse of Japanese Multiple Sclerosis

**DOI:** 10.5402/2011/904706

**Published:** 2011-09-06

**Authors:** Yoko Warabi

**Affiliations:** Department of Neurology, Tokyo Metropolitan Neurological Hospital, 2-6-1 Musashidai Fuchu, Tokyo 183-0042, Japan

## Abstract

For the purpose of predicting multiple sclerosis (MS) and neuromyelitis optica (NMO) relapses in Japanese population, we evaluated the localization and age of each demyelinating attack. We retrospectively analyzed the 78 medical records of Japanese MS and NMO patients. Then we identified 49 cases of relapsing-remitting-type patients and defined each of 116 demyelinating attacks. NMO had an older age at onset than MS, although the initial symptoms cannot predict the clinical phenotypes. Only 21.3% of demyelinating attacks were localized in the cerebrum and 78.7% were optic-spinal lesions, although MS comprised 70% and NMO comprised 30% of these 78 cases. Brainstem lesion had a relative male predominancy and a young age at attack. Our findings showed that optic nerve and spinal cord lesions are the major and critical lesions in each attack of Japanese CNS demyelinating diseases. There might be distinctive Japanese pathogenic features even in Western type MS.

## 1. Introduction

Multiple sclerosis (MS) and neuromyelitis optica (NMO) are inflammatory demyelinating diseases of the central nervous system (CNS). Although MS and NMO are characterized by dissemination of lesions in space and dissemination in time, it has not been able to predict when and where the next relapse will appear. One of the possibilities that suggests where the next relapse occurs is due to the clinical phenotype of the CNS inflammatory demyelinating diseases in the Japanese population. Japanese CNS demyelinating disease has two major clinical phenotypes. One is NMO [1, 2] which is characterized by lesions confined to the optic nerve and spinal cord, especially, longitudinally extensive spinal cord lesions [3], positive aquaporin-4 (AQP4) autoantibody seropositivity [4], and the astrocytic impairment associated with the loss of AQP4 in the NMO lesions [5]. The other is conventional Western type MS which is known to be the same pathophysiology as MS in western countries and frequently involves cerebral lesions [6]. Human leukocyte antigen (HLA)-DR2 antigen is reported to associate with MS both in Japan and western countries [7], whereas DPB1∗0501 is associated with NMO in Asian population [8, 9]. There is a consensus that NMO comprises about one-third or one-fourth of the Japanese CNS inflammatory demyelinating diseases [10]. 

In this paper, for the purpose of predicting the lesions of the demyelinating relapses, we evaluated the localization, sex ratio, and age of each demyelinating attack, retrospectively. As the results, we found that the attacks in optic nerve and spinal cord accounted for about 80% of all MS and NMO attacks, although NMO comprised only 30% of the Japanese CNS inflammatory demyelinating diseases. Furthermore, relapses in brainstem demonstrate unique characteristics compared to other lesions. Thus, optic nerve and spinal cord still play key roles in the Japanese CNS inflammatory demyelinating diseases of both clinical phenotypes of MS and NMO. 

## 2. Patients and Methods

### 2.1. Patients and Clinical Evaluation

We retrospectively analyzed the medical records of Japanese patients admitted to the Department of Neurology, Tokyo Metropolitan Neurological Hospital, for the first time between January 1992 and December 2001 and identified 78 patients diagnosed as having MS and NMO [1, 11], and we analyzed their baseline clinical characteristics. Then we followed them up and identified 49 cases of relapsing-remitting-type MS and NMO. We defined each of 116 clinical demyelinating attacks that were confirmed on MRI and optic neuritis diagnosed by ophthalmologists. Thirty-three attacks occurred in 15 NMO patients, and 83 attacks occurred in 34 MS patients.

### 2.2. Statistical Analysis

Student's *t*-test or Welch's *t*-test was used for the statistical analysis of the age, duration of disease, and EDSS. The sex ratio was analyzed using the chi-square test or Fisher's exact probability method (where appropriate). All statistical analyses were performed using a commercial software package (SPSS version 10.0J, SPSS Japan Inc., Tokyo, Japan).

## 3. Results

### 3.1. Clinical Characteristics of Japanese CNS Demyelinating Disease: Initial Symptoms Cannot Predict the Clinical Phenotypes

Dividing these 78 Japanese CNS demyelinating disease patients into the two clinical phenotypes, NMO comprised 29.5% and MS comprised 70.5%, as previously reported [10]. Table 1 shows the clinical characteristics of all the 78 Japanese CNS demyelinating disease patients. Age at onset was significantly older in NMO than in MS (*P* = 0.005). Sex ratio was not different between MS and NMO. Two major initial symptoms were sensory disturbance and visual loss both in MS and NMO. Other symptoms did not have enough number to evidently discuss. Thus, in this analysis in a general view, older age at onset is associated with the larger possibility to be classified to NMO, although sex ratio and the initial symptoms cannot predict the clinical phenotypes in Japanese population. 

### 3.2. Localization of Each Clinical Demyelinating Attack: Optic Nerve and Spinal Cord Were the Major Lesions in Japanese MS

**Figure 1:**
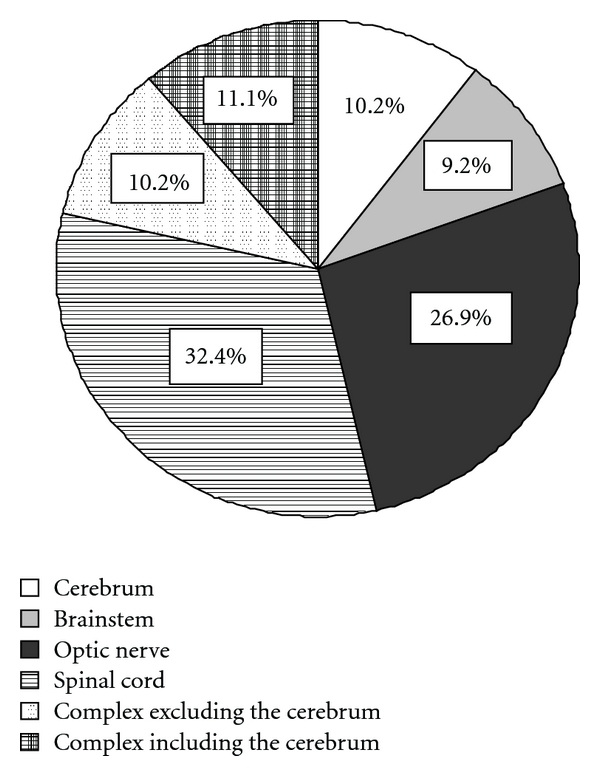
Localization of each of 116 demyelinating attacks from 49 relapsing-remitting MS and NMO patients. 10.2% involved the cerebrum, 9.2% the brainstem, 26.9% the optic nerve, 32.4% the spinal cord, 10.2% involved complex lesions excluding the cerebrum and, 11.1% complex lesions including the cerebrum. Thus, only 21.3% of demyelinating attacks were localized in cerebrum and 78.7% were optic-spinal lesions, although the clinical phenotype showed that MS comprised 70% and NMO comprised 30%.

Localization of each of these 116 demyelinating attacks from 49 relapsing-remitting-type MS and NMO patients is shown in Figure 1. Overall, 10.2% occurred in the cerebrum, 9.2% the brainstem, 26.9% the optic nerve, 32.4% spinal cord, 10.2% comprised complex lesions excluding the cerebrum, and 11.1% complex lesions including the cerebrum. Thus, only 21.3% of demyelinating attacks were localized in the cerebrum and 78.7% were optic-spinal lesions, although the clinical phenotype showed that 70% occurred in MS and 30% in NMO.

### 3.3. Brainstem Lesion Has a Relative Male Predominancy and a Young Age at Attack

**Figure 2:**
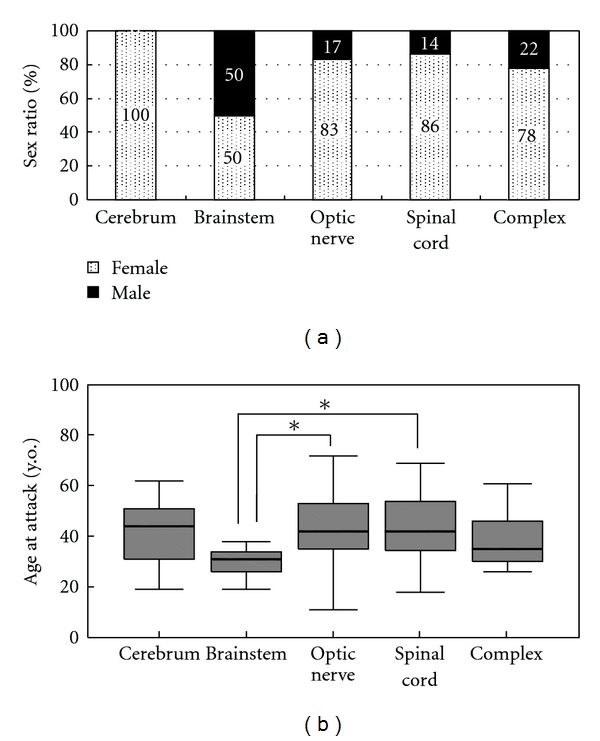
(a) Sex ratio of each localization of MS attacks. Half of the brainstem lesions occurred in males, although there was a remarkable female predominancy for other lesion sites. (b) Age at attack for each localization of MS attacks. Mean age at brainstem attack was 29.6  ±  6.0 years old and is significantly younger than the mean age at optic nerve attack (42.5  ±  14.4 y.o.) and spinal cord attack (42.6  ±  13.6 y.o.) using Welch's *t*-test (*P*  <  0.05). Regarding other lesions, mean patient age at attacks involving the cerebrum was 42.3  ±  13.5 y.o., and the mean age at the development of complex lesions was 38.7  ±  10.9 y.o. Boxes represent values from the 25th to the 75th percentiles, inner lines represent the median, and whiskers show the minimal and maximal values.

The characteristics of each demyelinating localization were further investigated. Figure 2(a) shows that half of the brainstem lesions occurred in males, although there was a remarkable female predominancy for other lesion sites. 

Furthermore, mean patient age at brainstem attack was 29.6  ±  6.0 year old and tended to be younger than that for other lesions. Especially, mean patient age at brainstem attack was significantly younger than that at optic nerve attack (42.5  ±  14.4 y.o.) or spinal cord attack (42.6  ±  13.6 y.o.) using Welch's *t*-test (*P *< 0.05) (Figure 2(b)). Brainstem attacks occurred mostly between 20 and 40 years old, although attacks at other localizations were widely distributed from teenage to over 60 years old.

## 4. Discussion

This analysis is unique because we examined each patient in detail characterizing each demyelinating attack, although the initial symptoms of each MS patients have been widely discussed. It was reported that visual loss is one of the most common initial symptoms in Japan, although the incidence of sensory and motor dysfunctions is larger than visual loss in the western countries [12, 13]. There had been adequate importance to assess the initial symptoms in the era of when MRI was not invented and the clinical diagnosis of MS was very difficult. However, our study showed that initial symptoms were no longer able to predict the relapses and the clinical phenotypes for each Japanese CNS demyelinating disease patients.

Our findings showed that optic nerve and spinal cord lesions were the major and critical lesions in each attack of Japanese CNS demyelinating diseases. Japanese MS has been considered the same disease entity as prototypic western MS. Moreover, recent revision of the McDonald Criteria for MS in 2010 clearly defined that once NMO and NMO spectrum disorders have been excluded, Western type MS in Asian population is not fundamentally different from typical MS in the Caucasian population [6]. However, our findings suggest that there might be distinctive Japanese pathogenic features even in MS, and, therefore, optic nerve attacks and spinal cord attacks remain to have critical roles. It might be possible that attacks causing visual loss or transverse myelitis are severe and require hospital care; therefore, the number of optic and spinal attacks was increased in this study. However, we think that this hypothesis is inaccurate because the mean EDSS did not differ between NMO and MS. 

Brainstem lesions demonstrate striking differences in sex ratio and age at relapse compared to other lesions and were frequently seen in young adult males. Although sex differences in the pathomechanism for MS are unsolved problems, this feature would be explained by hormonal and genetic studies [14]. 

Interferon beta-1b (IFNB-1b) was reported to be effective for not only patients in Western countries but also Japanese relapsing-remitting MS patients according to a randomized, double-blind clinical study [15]. Conversely, IFNB-1b treatment for NMO could increase the relapse rate of NMO or cause severe side effects such as severe skin ulcers at injection sites requiring surgical repair; thus, it should not be administered to NMO patients [16]. We suspect that certain Japanese MS patients whose optic nerve attacks and spinal cord attacks have critical roles might play an intermediate role between MS and NMO and that the treatment adequate for those intermediate patients might be not only immunomodulating therapy including IFNB-1b but also immunosuppressive therapy including prednisolone, immunosuppressant, monoclonal antibodies, and biologics.

## 5. Conclusions

Our findings showed that optic nerve and spinal cord lesions were the major and critical lesions in each attack of Japanese MS. There might be the Japanese distinctive pathogenic features even in Western type MS, and, therefore, optic nerve attacks and spinal cord attacks remain to have critical roles in the Japanese population. We suspect that certain Japanese MS patients might play an intermediate role between MS and NMO and that the treatment adequate for those intermediate patients might be not only immunomodulating therapy but also immunosuppressive therapy. 

## Figures and Tables

**Table 1:** Clinical characteristics and initial symptoms of MS and NMO patients.

Characteristics	Total of MS and NMO	MS	NMO
	*n* = 78	*n* = 55	*n* = 23
Sex			
Male/female	21 (27%)/57 (73%)	15 (27%)/40 (73%)	6 (26%)/17 (74%)
Age at onset, y.o.			
Mean ± SD	35.3 ± 13.6	32.5 ± 13.0^a^	41.9 ± 13.1^a^
Duration of disease, years			
Mean ± SD	5.2 ± 7.0	6.0 ± 7.1	3.3 ± 6.3
EDSS			
Mean ± SD	3.9 ± 2.5	3.9 ± 2.6	3.9 ± 2.2
Initial symptoms			
Sensory disturbance	29%	24%	44%
Visual loss	26%	27%	22%
Double vision or vertigo	12%	15%	4%
Acute motor weakness	5%	5%	4%
Chronic motor weakness	5%	7%	0%
Sphincter disturbance	4%	2%	9%
Ataxia	1%	2%	0%
Consciousness disturbance	1%	2%	0%
Complex	17%	16%	17%

^a^Mean age at onset of NMO patients was significantly older than that of MS patients (*P* = 0.005). Student's *t*-test was used for the statistical analysis.
